# Imaging-based body fat distribution and diabetic retinopathy in general US population with diabetes: an NHANES analysis (2003–2006 and 2011–2018)

**DOI:** 10.1038/s41387-024-00308-z

**Published:** 2024-07-14

**Authors:** Chenxin Li, Yili Zhang, Yujie Wang, Chufeng Gu, Bo Li, Mingming Ma, Xiaoyin Xu, Yongdong Chen, Zhi Zheng

**Affiliations:** 1 Department of Ophthalmology, Shanghai General Hospital, Shanghai Jiao Tong University School of Medicine, National Clinical Research Center for Eye Diseases, Shanghai Clinical Research Center for Eye Diseases, Shanghai Key Clinical Specialty, Shanghai Key Laboratory of Ocular Fundus Diseases, Shanghai Engineering Center for Visual Science and Photomedicine, Shanghai Engineering Center for Precise Diagnosis and Treatment of Eye Diseases, Shanghai, 200080 China; 2 Department of Ophthalmology, Shanghai General Hospital, Shanghai, 200080 China; 3 Centre for Eye Research Australia, Royal Victorian Eye and Ear Hospital, Melbourne, Australia; 4 Ophthalmology, Department of Surgery, University of Melbourne, Melbourne, Australia; 5 Department of Ophthalmology, Renji Hospital, Shanghai Jiao Tong University School of Medicine, Shanghai, 200127 China; 6 Ningde Municipal Hospital, Ningde Normal University, Ningde, China; 7 Fujian Medical University, Fuzhou, China

**Keywords:** Risk factors, Epidemiology

## Abstract

**Background:**

Limited studies have investigated the correlation between fat distribution and the risk of diabetic retinopathy (DR) in the general population with diabetes. The relationship between obesity and DR remains inconclusive, possibly due to using simple anthropometric measures to define obesity. This study investigates the relationships between the android-to-gynoid fat ratio (A/G ratio, measured using dual-energy X-ray absorptiometry) and DR within the US population with diabetes.

**Methods:**

The study used a population-based, cross-sectional approach based on the 2003–2006 and 2011–2018 data of the National Health and Nutrition Examination Survey (NHANES). Multivariable logistic regression analyses were performed on participants with diabetes to evaluate the contribution of body mass index (BMI), waist-to-height ratio (WHtR), and A/G ratio to the prevalence of DR.

**Results:**

The prevalence of DR was 22.2, 21.2, and 17.6% among participants with A/G ratios <1.0, 1.0–1.2, and ≥1.2, respectively. After adjusting sex, age, ethnicity, diabetes duration, hemoglobin A1c level, blood pressure level, and non-high-density lipoprotein cholesterol level, a higher A/G ratio (≥1.2) was independently associated with decreased odds of DR (odds ratio [OR], 0.565; 95% CI: 0.372–0.858) compared with the A/G ratio of 1.0–1.2. Associations between a higher A/G ratio and DR remained statistically significant after adjusting for BMI (OR, 0.567; 95% CI: 0.373–0.861) and WHtR (OR, 0.586; 95% CI: 0.379–0.907). Moreover, these associations remained statistically significant in analyses using the ethnic-specific tertiles for the A/G ratio. In sex-stratified models, these correlations remained in males. There was a significant inverse association between the A/G ratio and diabetes duration in males, which persisted after multivariable adjustments (*p* < 0.05).

**Conclusions:**

A novel finding indicates that a higher A/G ratio is associated with a reduced likelihood of DR in males with diabetes. The results from NHANES underscore the importance of considering imaging-based fat distribution as a critical indicator in clinical practice.

## Introduction

The prevalence of diabetes mellitus (DM) is increasing worldwide, with a projected 46% surge by the year 2045 [1]. Diabetic retinopathy (DR) is a primary microvascular complication of DM and is a leading cause of vision impairment and blindness worldwide [2]. Studies have implicated a prevalence of DR at 37% among newly diagnosed diabetics [3, 4]. Given the escalating global prevalence of diabetes, the number of individuals with DR is expected to grow, further exacerbating the negative social impact of this condition.

Obesity is a well-recognized risk factor for DM [5]. While obesity has been linked to increased risks of cardiovascular complications and kidney disease in individuals with diabetes [6], its associations with diabetic retinal complications have yielded inconclusive findings. Various studies have reported mixed results regarding the relationships between body mass index (BMI) and DR. A study indicated that a higher BMI was associated with DR [7]. The Diabetes Incidence Study in Sweden found a significant association between baseline high BMI and severe non-proliferative DR and proliferative DR over a 10-year follow-up [8]. The similar associations were reported in the World Health Organization (WHO) study [9] and in the Diabetes Control and Complications Trial [10]. Conversely, the Singapore Diabetes Management Project [11] and other studies [12–15] have suggested that a higher BMI may confer a protective effect against DR. Moreover, the Wisconsin Epidemiologic Study of Diabetic Retinopathy found no significant association between obesity (defined by BMI) and the risk of DR incidence or progression in type 2 DM (T2DM) [16]. Likewise, the Hoorn Study found no significant association between BMI and DR incidence in T2DM individuals [17]. Similarly, inconsistent results were observed for the associations between DR and waist-to-hip ratio (WHR) and waist-to-height ratio (WHtR) in some studies [7, 13, 18–20]. The discrepancies in the reported association between BMI, WHR, or WHtR, and DR may be partially attributable to the use of simple anthropometric measures to define obesity, which may not fully reflect body fat accumulation and associated risk factors. Consequently, further research is necessary to better understand the role of body fat deposits in the development and progression of DR.

Substantial evidence supports causal relationships between excess fat in specific body regions and metabolic and cardiovascular disease [21–25]. The gynoid fat pattern, characterized by preferential fat deposition in the hip and thigh regions, has demonstrated a protective effect against cardiovascular and diabetes-related mortality and impaired glucose metabolism. Conversely, the android fat pattern, consisting primarily of adipose tissue around the trunk, is associated with the development of cardiovascular risk factors [26]. Dual-energy X-ray absorptiometry (DXA) is a well-established, non-invasive method for measuring body composition with minimal radiation exposure. It provides an accurate assessment of regional adipose tissue depots, allowing for the quantification of fat distribution, including android and gynoid fat masses. Among its clinical utilities, the android-to-gynoid fat ratio (A/G ratio) is reported to be associated with incident T2DM [27], insulin resistance [28], nonalcoholic fatty liver disease [29], atherosclerosis [30, 31], and atrial fibrillation [32]. Despite the clinical utility of DXA in evaluating fat distribution and its links to various health conditions, limited studies have investigated the impact of fat distribution on retinopathy in the population with diabetes. Consequently, the current study aims to fill this gap and determine the contribution of specific regional fat accumulation patterns to DR in individuals with diabetes. The DXA-assessed body composition data will be used to investigate the independent role of body fat distribution in the prevalence of DR.

## Methods

### Data sources

This study has presented an analysis of data obtained during the 2003–2004, 2005–2006, 2011–2012, 2013–2014, 2015–2016, and 2017–2018 cycles of the National Health and Nutrition Examination Survey (NHANES). NHANES is a complex cross-sectional survey program conducted in the United States (U.S.) by the National Center for Health Statistics (NCHS) of the Center for Disease Control and Prevention (https://www.cdc.gov/nchs/nhanes/index.htm). NHANES conducted a complex, multistage, clustered probability sampling design and included a representative sample of the general US population of all ages. NHANES adhered to the principles of the Declaration of Helsinki, and its research procedures were approved by the NCHS Research Ethics Review Board. Informed consent was obtained from all survey participants. Ethical approval was not required for this study because the NCHS Research Ethics Review Board approved the NHANES protocols. The investigation was based on secondary analyses of de-identified data.

### Study design and analytic sample

**Fig. 1:**
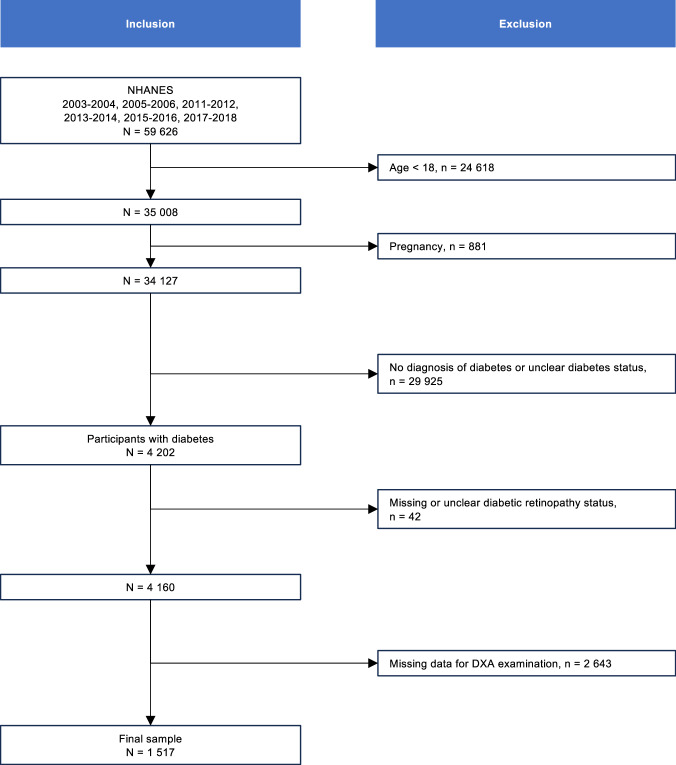
Flow chart of the study population. A flow chart presents the inclusion and exclusion criteria for the study. *NHANES* National Health and Nutrition Examination Survey, *DXA* dual-energy X-ray absorptiometry.

The study included participants in six NHANES cycles, covering 2003–2004, 2005–2006, 2011–2012, 2013–2014, 2015–2016, and 2017–2018. Diabetes was defined as a self-report of diabetes diagnosis by a physician or other health professional [33]. The study population included participants with diabetes. Pregnant participants, individuals aged <18 years, individuals without information for DR, or those with invalid A/G ratio values were excluded from the analysis, leading to a final sample of 1517 participants with available data (Fig. 1).

### Study variables

Demographic variables included in this study were age, gender, and race/ethnicity. The duration of diabetes and diabetic retinopathy were extracted from diabetes questionnaires [33–36].

Weight, height, waist circumference, and blood pressure readings were collected in mobile examination centers with standard protocols. BMI was calculated as weight in kilograms divided by height in meters squared and was classified as <25, 25–30, and ≥30 kg/m^2^, according to the WHO definition for international BMI classification. The WHtR was determined by dividing the waist circumference by height and was categorized into three tertiles based on the data of the final sample population: the first tertile (<0.61), the second tertile (0.61–0.69), and the third tertile (≥0.69).

The A/G ratio was measured by DXA. Android and gynoid regions were defined by the Hologic APEX software used in the scan analysis [37]. Tertiles of the data from the final sample population were utilized to classify the A/G ratio into the following: first tertile (<1.0), second tertile (1.0–1.2), and third tertile (≥1.2).

Non-high-density lipoprotein cholesterol (non-HDL-C), a measure of total cholesterol minus high-density lipoprotein cholesterol, was used as a measure of dyslipidemia in analyses. Non-HDL-C was demonstrated to be a predictor of cardiovascular disease, and individuals with diabetes were recommended to achieve a non-HDL-C target of <130 mg/dL [38, 39]. Glycemic control was assessed by hemoglobin A1c (HbA1c) and was stratified into well-controlled (HbA1c < 53 mmol/mol [7.0%]) and poorly controlled (HbA1c ≥ 53 mmol/mol [7.0%]). Controlled hypertension was set as blood pressure less than 140/90 mmHg. Detailed descriptions of blood collection and processing procedures were provided on the NHANES website.

### Data analysis

In sensitivity analyses, the study population was partitioned into tertiles according to ethnic subgroups in consideration of racial and ethnic variations in BMI, WHtR, and the A/G ratio. BMI was analyzed continuously and categorically (using the international WHO definitions for generalized obesity and tertiles defined within each ethnic subgroup). WHtR and the A/G ratio were assessed continuously and categorically in tertiles (using both the overall sample population tertiles and ethnic subgroup tertiles). Meanwhile, the analyses were repeated after stratifying participants by sex. To test the robustness of the findings, possible individuals with type 1 DM (T1DM) were excluded (defined as those aged <20 years who used only insulin) [34, 40] and sensitivity analyses were performed on a subgroup of participants with T2DM. Additionally, the analyses were repeated after further adjustment for insulin use in the subgroup of participants with T2DM.

Analyses were performed in RStudio for macOS (2022.02.0 + 443) using the R Package Survey (4.1–1). We used appropriate NHANES weights to account for the complex survey design of NHANES. Baseline characteristics of the participants were expressed as medians with interquartile ranges (M (P25, P75)) and means with standard deviations (mean (SD)) for continuous variables and as percentages (*n* (%)) for categorical variables. The Mann–Whitney *U*-test and the Rao-Scott chi-square test were used for continuous and categorical variables, respectively. Multivariable logistic regression analyses were performed to assess the associations between BMI, WHtR, and the A/G ratio with the presence of DR, respectively, adjusted for potential confounding factors established in previous research. These variables included age, sex, ethnicity, diabetes duration, HbA1c level, blood pressure level, and non-high-density lipoprotein cholesterol level. Multivariable linear regression analysis assessed the associations between diabetes duration and the A/G ratio. A two-sided *P* value of <0.05 was used to determine statistical significance.

## Results

### Study population characteristics

The study sample included 1517 participants, and the estimated population size was 9,019,027 individuals. The study population contained 752 females (49.6%), and the median age (interquartile range) of the study participants was 52 (44, 57) years. Table 1 reports the demographic and clinical characteristics of the participants stratified by presence/absence of DR. The prevalence of DR was 20.0% among the population with diabetes included in the study. Compared with participants without DR, participants with DR were likely to have a longer duration of diabetes and a higher HbA1c level (both *P* < 0.05).

**Table 1:** Weighted descriptive statistics of the US population with diagnosed diabetes stratified by the presence of diabetic retinopathy.

Variable	Overall (*N* = 1 517)	No DR (*N* = 1 207)	DR (*N* = 310)	*P* value
**Weighted** ***N*, %**	9 019 027	7 214 811 (80.0%)	1 804 216 (20.0%)	
**Age (years, mean [SD])**	51.3 (11.42)	51.2 (11.65)	51.8 (10.43)	
**Age (years, median [IQR])**	52 (44,57)	52 (44,57)	52 (46,57)	0.468
**Gender, %** ^a^				
male	765 (50.4%)	593 (48.8%)	172 (56.6%)	0.070
female	752 (49.6%)	614 (51.2%)	138 (43.4%)	
**Race/ethnicity, %** ^a^				
Mexican American	351 (11.7%)	275 (11.9%)	76 (10.9%)	0.597
Other Hispanic	109 (7.0%)	88 (7.3%)	21 (5.9%)	
Non-Hispanic White	460 (55.6%)	369 (55.7%)	91 (55.3%)	
Non-Hispanic Black	408 (16.5%)	330 (16.6%)	78 (16.0%)	
Other race	189 (9.3%)	145 (8.6%)	44 (11.9%)	
**DM duration (years, %** ^a^**)**				
<10	908 (63.0%)	770 (68.4%)	138 (41.2%)	<0.001
10–20	391 (23.0%)	304 (22.3%)	87 (25.9%)	
≥20	212 (14.0%)	127 (9.3%)	85 (32.9%)	
**HbA1c level, %** ^a^				
<53 mmol/mol (7%)	696 (50.6%)	586 (54.0%)	110 (37.1%)	<0.001
≥53 mmol/mol (7%)	779 (49.4%)	592 (46.0%)	187 (62.9%)	
**Blood pressure level (mmHg, %** ^a^**)**				
<140/90	1 094 (78.4%)	889 (79.3%)	205 (74.6%)	0.204
≥140/90	363 (21.6%)	272 (20.7%)	91 (25.4%)	
**Non-HDL-C level (mg/dL, %** ^a^**)**				
<130	610 (44.0%)	484 (42.8%)	126 (48.7%)	0.245
≥130	844 (56.0%)	677 (57.2%)	167 (51.3%)	
**BMI (kg/m**^**2**^**, mean [SD])**	32.7 (7.30)	32.8 (7.17)	32.0 (7.79)	
**BMI (kg/m**^**2**^**, median [IQR])**	31.5 (27.5,37.0)	31.7 (27.8,37.1)	31.3 (26.3,36.5)	0.199
**BMI level, %** ^a^				
<25.0	213 (12.7%)	157 (12.1%)	56 (14.9%)	0.525
25.0 ~ 30.0	449 (27.1%)	365 (26.8%)	84 (28.3%)	
≥30.0	847 (60.2%)	678 (61.0%)	169 (56.8%)	
**WHtR (mean [SD])**	0.65 (0.10)	0.65 (0.10)	0.64 (0.11)	
**WHtR (median [IQR])**	0.64 (0.58,0.71)	0.64 (0.58,0.71)	0.64 (0.57,0.71)	0.333
**WHtR categories, %** ^a^				
Tertile 1 (<0.61)	553 (34.9%)	439 (34.2%)	114 (37.9%)	0.693
Tertile 2 (0.61–0.69)	495 (32.5%)	397 (32.8%)	98 (31.5%)	
Tertile 3 (≥0.69)	443 (32.6%)	351 (33.1%)	92 (30.7%)	
**A/G ratio (mean [SD])**	1.1 (0.19)	1.1 (0.19)	1.1 (0.21)	
**A/G ratio (median [IQR])**	1.1 (1.0,1.2)	1.1 (1.0,1.2)	1.1 (1.0,1.2)	0.416
**A/G ratio categories, %** ^a^				
Tertile 1 (<1.0)	286 (18.6%)	226 (18.1%)	60 (20.6%)	0.421
Tertile 2 (1.0–1.2)	656 (43.1%)	517 (42.5%)	139 (45.6%)	
Tertile 3 (≥1.2)	575 (38.3%)	464 (39.4%)	111 (33.7%)	

*P* value was calculated by the Mann–Whitney *U*-test for continuous variables and the Rao-Scott chi-square test for categorical variables.

SI conversion factors: To convert non-high-density lipoprotein cholesterol to mmol/L, multiply values by 0.02586.

*Non-HDL-C* non-high-density lipoprotein cholesterol, *DM* diabetes mellitus, *HbA1c* hemoglobin A1c, *BMI* body mass index, *WHtR* waist-to-height ratio, *A/G ratio* android-to-gynoid fat ratio.

^a^Unweighted counts (weighted percent) for categorical variables.

### Associations of BMI and WHtR with diabetic retinopathy were not evident

Table 2 shows the associations between BMI, WHtR, and DR. BMI was analyzed as a continuous variable or categorical variable using WHO standards and defined tertiles in ethnic subgroups. The associations between BMI and DR were not evident in multivariable adjustments, including sex, age, ethnicity, diabetes duration, HbA1c level, blood pressure level, and non-high-density lipoprotein cholesterol level. Sex-stratified analyses found that females in the population with BMI (25.0–30.0 kg/m^2^) were less likely to have DR compared with females in the population with BMI <25.0 kg/m^2^ (OR, 0.447; 95% CI: 0.212–0.943). The sex-stratified analysis using the ethnic-specific tertiles for BMI did not show similar significant results in females. Sex-stratified analyses failed to reveal any significant association between BMI and DR in males.

**Table 2:** Multivariable associations between BMI and waist-to-height ratio with the presence of diabetic retinopathy in the studied population and stratified by sex.

	Prevalence	Overall OR (95% CI)	Male OR (95% CI)	Female OR (95% CI)
**BMI level**				
**<25**	23.6%	1 [Reference]	1 [Reference]	1 [Reference]
≥**25–**<**30**	20.9%	0.908 (0.506, 1.630)	1.270 (0.586, 2.751)	0.447 (0.212, 0.943)
≥**30**	18.9%	0.957 (0.537, 1.705)	1.113 (0.527, 2.352)	0.671 (0.299, 1.507)
***P*** **for trend**		0.970	0.954	0.742
**BMI tertiles by ethnicity**				
**Tertile 1**	24.2%	1 [Reference]	1 [Reference]	1 [Reference]
**Tertile 2**	18.0%	0.817 (0.535, 1.247)	0.774 (0.426, 1.403)	0.897 (0.451, 1.782)
**Tertile 3**	18.0%	0.836 (0.520, 1.342)	0.734 (0.388, 1.389)	0.903 (0.456, 1.785)
***P*** **for trend**		0.445	0.314	0.779
**BMI (Per unit increase)**		1.001 (0.973, 1.030)	0.995 (0.956, 1.036)	1.004 (0.965, 1.044)
**WHtR level**				
**Tertile 1 (**<**0.61)**	21.5%	1 [Reference]	1 [Reference]	1 [Reference]
**Tertile 2 (0.61–0.69)**	19.1%	1.116 (0.713, 1.748)	1.109 (0.628, 1.958)	1.153 (0.477, 2.785)
**Tertile 3 (**≥**0.69)**	18.6%	1.037 (0.650, 1.654)	0.949 (0.468, 1.925)	1.161 (0.510, 2.643)
***P*** **for trend**		0.859	0.951	0.728
**WHtR tertiles by ethnicity**				
**Tertile 1**	21.4%	1 [Reference]	1 [Reference]	1 [Reference]
**Tertile 2**	18.8%	1.078 (0.702, 1.654)	1.090 (0.625, 1.899)	1.144 (0.512, 2.554)
**Tertile 3**	19.1%	1.030 (0.649, 1.634)	0.887 (0.447, 1.757)	1.217 (0.540, 2.740)
***P*** **for trend**		0.892	0.792	0.634
**WHtR (Per 0.1-unit increase)**		1.003 (0.813, 1.239)	1.000 (0.748, 1.337)	1.009 (0.730, 1.393)

Adjusted for sex, age, race/ethnicity, diabetes duration, hemoglobin A1c level, blood pressure level, non-high-density lipoprotein cholesterol level.

Stratified models are adjusted for covariates not stratified on.

*BMI* body mass index, *WHtR* waist-to-height ratio, *OR* odds ratio, *CI* confidence interval.

In multivariable-adjusted and sex-stratified models, continuous or categorical analysis of WHtR failed to reveal any significant associations for DR (all *P* > 0.05). Analyses conducted on the population with T2DM produced the same results as those with all diabetes (Table S1).

### Higher A/G ratio exerts a protective effect against DR

For evaluating the contribution of fat distribution to the risk of DR, the A/G ratio was categorized by tertiles, and multivariable logistic regression was performed (Table 3), adjusting sex, age, ethnicity, diabetes duration, HbA1c level, blood pressure level, and non-HDL-C level. The prevalence of DR was 22.2, 21.2, and 17.6% among the populations with A/G ratio <1.0, 1.0–1.2, ≥1.2, respectively. The prevalence of DR was 21.2, 21.6, and 17.8% among the populations in tertile one, tertile two, and tertile three, respectively, when using ethnic-specific tertiles.

**Table 3:** Multivariable associations between A/G ratio and the presence of diabetic retinopathy.

A/G ratio	Prevalence (%)	Odds ratio (95% CI)		
		Model 1^a^	Model 2^b^	Model 3^c^
**Tertiles categorized by overall population**				
**1.0–1.2**	21.2%	1 [Reference]	1 [Reference]	1 [Reference]
**<1.0**	22.2%	0.813 (0.485, 1.362)	0.831 (0.487, 1.417)	0.877 (0.514, 1.496)
**≥1.2**	17.6%	0.565 (0.372, 0.858)	0.567 (0.373, 0.861)	0.586 (0.379, 0.907)
***P*** **for trend**		0.061	0.047	0.046
**Tertiles categorized by ethnicity**				
**Tertile 2**	21.6%	1 [Reference]	1 [Reference]	1 [Reference]
**Tertile 1**	21.2%	0.768 (0.452, 1.304)	0.776 (0.450, 1.339)	0.815 (0.471, 1.411)
**Tertile 3**	17.8%	0.570 (0.369, 0.880)	0.573 (0.372, 0.884)	0.594 (0.377, 0.936)
***P*** **for trend**		0.106	0.088	0.087
**A/G ratio (per 0.1-unit increase)**		0.934 (0.834, 1.046)	0.926 (0.821, 1.044)	0.922 (0.810, 1.049)

^a^Model 1: Adjusted for sex, age, race/ethnicity, diabetes duration, hemoglobin A1c level, blood pressure level, non-high-density lipoprotein cholesterol level.

^b^Model 2: Model 1+body mass index.

^c^Model 3: Model 1+waist-to-height ratio.

*A/G ratio* android-to-gynoid fat ratio, *OR* odds ratio, *CI* confidence interval.

After adjusting for several covariates, the population in the highest tertile of A/G ratio (≥1.2) were less likely to have DR (OR, 0.565; 95% CI: 0.372–0.858) compared with those in the middle A/G ratio tertile (1.0–1.2). After further adjustment for BMI, the association between the A/G ratio and DR was unattenuated (OR, 0.567; 95% CI: 0.373–0.861). Moreover, the test for trend was significant (*P* for trend = 0.047). After adjusting WHtR, the association between the A/G ratio and DR persisted (OR, 0.586; 95% CI: 0.379–0.907), and the test for trend was significant (*P* for trend = 0.046). The analysis using the ethnic-specific tertiles for the A/G ratio, found that the individuals in the highest tertile of the A/G ratio were less likely to have DR (OR, 0.570; 95% CI: 0.369–0.880) compared with those in the middle A/G ratio tertile. The associations persisted after further adjustment for BMI (OR, 0.573; 95% CI: 0.372–0.884) and WHtR (OR, 0.594; 95% CI: 0.377–0.936). Moreover, analyses based on the population with T2DM yielded the same results as those with all diabetes (Table S2).

**Fig. 2:**
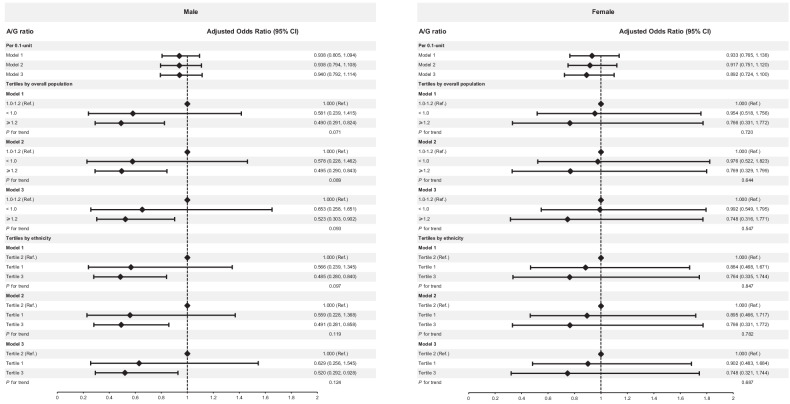
Multivariable associations between A/G ratio and presence of diabetic retinopathy, stratified by sex. Values are shown as odds ratios (OR) and 95% confidence interval (95%CI). The model was adjusted for age, race/ethnicity, diabetes duration, hemoglobin A1c level, blood pressure level, and non-high-density lipoprotein cholesterol level. Model 1: Adjusted for age, race/ethnicity, diabetes duration, hemoglobin A1c level, blood pressure level, and non-high-density lipoprotein cholesterol level. Model 2: Model 1+body mass index. Model 3: Model 1+waist-to-height ratio.

In sex-stratified analyses (Fig. 2), compared to the middle A/G ratio (1.0–1.2), a higher A/G ratio (≥1.2) was associated with lower odds of DR in males with diabetes (OR, 0.490; 95% CI: 0.291–0.824), but not in females. The associations persisted after adjustment for BMI (OR, 0.495; 95% CI: 0.290–0.843) in males. Meanwhile, the associations remained after adjusting WHtR (OR, 0.523; 95% CI: 0.303–0.902) in males. The sex-stratified analysis using the ethnic-specific tertiles for the A/G ratio found that the male individuals in the highest tertile of the A/G ratio were less likely to have DR (OR, 0.485; 95% CI, 0.280–0.840) compared with those in the middle A/G ratio tertile. The associations persisted after further adjustment for BMI (OR, 0.491; 95% CI: 0.281–0.858) and WHtR (OR, 0.520; 95% CI: 0.292–0.928). The sex-stratified analyses failed to reveal any significant associations for DR (all *P* > 0.05) in female individuals. The studies based on the population with T2DM found similar effective results as those with all diabetes (Table S3). Additionally, the associations remained significant after further adjustment for insulin use within the population with T2DM (Table S4).

### The A/G ratio exhibits a negative correlation with diabetes duration

Table 4 depicts the linear associations between fat depots and diabetes duration. After adjustment for demographic factors, the coefficient for diabetes duration suggested that a per-10-year increase in DM was associated with a 0.018 decrease in the A/G ratio (*p* = 0.004). Similar inverse associations were observed after additional adjustments (Table 4; models 2 and 3). In sex-stratified analyses, a per-10-year increase in DM was associated with a 0.026 decrease in the A/G ratio among the male population (*p* = 0.005). The inverse relationship persisted after multivariable adjustments (Table 4; models 2 and 3). The association was non-significant in the female population.

**Table 4:** Linear regression for association between A/G ratio and diabetes duration in the studied population and stratified by sex.

A/G ratio	DM duration (per-10-year increment)					
	Overall		Male		Female	
	*Beta* (95% CI)^*1*^	*P* value	*Beta* (95% CI)^*1*^	*P* value	*Beta* (95% CI)^*1*^	*P* value
**Model 1**	−0.018 (−0.030, −0.006)	0.004	−0.026 (−0.043, −0.008)	0.005	−0.011 (−0.030, 0.008)	0.250
**Model 2**	−0.016 (−0.028, −0.004)	0.009	−0.022 (−0.039, −0.004)	0.018	−0.013 (−0.033, 0.008)	0.217
**Model 3**	−0.012 (−0.024, 0.000)	0.056	−0.019 (−0.037, 0.000)	0.045	−0.006 (−0.024, 0.012)	0.527

Model 1: Adjusted for age, sex, race/ethnicity.

Model 2: Model 1+ hemoglobin A1c level.

Model 3: Model 2+ blood pressure level, non-high-density lipoprotein cholesterol level.

Stratified models are adjusted for covariates not stratified on.

*DM* diabetes mellitus, *CI* confidence interval.

## Discussion

In this population-based cross-sectional study, we investigated the associations between body fat distribution, as assessed by DXA, and the prevalence of DR. This study has presented new information that the higher A/G ratio is associated with a reduced likelihood of DR presence, independent of BMI and WHtR, although the A/G ratio-DR association is attenuated in females. The results provided valuable insights by highlighting the associations between imaging-based body fat distribution and DR, even after accounting for anthropometric obesity indicators, indicating that fat distribution may play an independent role in the pathophysiology of DR in the population with diabetes.

The association between obesity and DR has been a subject of debate for decades, with inconclusive evidence supporting the relationship between obesity indicators and DR. Studies have reported conflicting results, with some indicating that obesity, measured by higher BMI, is a significant independent risk factor for developing DR [7–10], while others have reported it to be protective [11–15], or have found no significant associations [16, 17] with DR. The associations between DR and WHtR were also inconsistent [19, 20]. To shed light on this relationship, the study evaluated the impact of anthropometric obesity indicators (BMI and WHtR) and body fat distribution evaluated by DXA on the prevalence of DR in the population of 1517 participants with diabetes from the NHANES. When analyzing BMI according to WHO standards, the results showed that overweight females with diabetes were less likely to have DR compared with females in the population with a BMI <25 kg/m^2^ (OR, 0.447; 95% CI: 0.212–0.943). However, no similar result was found in female participants when using the ethnic-specific tertiles for BMI, and no significant association between BMI and DR was found among males. Moreover, no significant association was found between WHtR and the prevalence of DR.

Interestingly, the study revealed that a higher A/G ratio was associated with lower DR prevalence, although the A/G ratio-DR association was attenuated in female participants. These associations persisted after adjusting for BMI and WHtR, suggesting that BMI or WHtR may not accurately estimate the risk of DR. The findings presented robust associations in sensitivity analyses using the ethnic-specific tertile for the A/G ratio. The study highlights the complexity of the relationship between obesity and DR and emphasizes the importance of considering other factors, such as body fat distribution, to better understand this association.

Android fat is the accumulation of adipose tissue around the trunk, while gynoid fat refers to the fat deposited in the subcutaneous depot of hips, thighs, and buttocks [21]. A higher A/G ratio is typically associated with an android pattern of fat accumulation, characterized by increased visceral adipose tissue (VAT) around the trunk or decreased subcutaneous adipose tissue (SAT) around the hips. Interestingly, subcutaneous fat, rather than VAT, has been reported to be associated with the site of inflammation onset in the elderly [32, 41]. Additionally, age-related telomere length shortening in SAT, not VAT, leads to an increase in senescent cell burden [41]. Given that individuals with DR are often older and that inflammation plays a role in the pathogenesis of microvascular complications, it is plausible that gynoid fat may pose a higher risk of DR in the elderly when compared to android fat. However, it is essential to acknowledge that the relationship between body fat distribution, inflammation, and aging is complex, and research is needed to fully understand their impact on DR risk.

Additionally, the current analyses of the A/G ratio in relation to the duration of DM revealed a significant association, indicating that a prolonged duration of DM was linked to a lower A/G ratio in males. This finding suggests that diabetes may alter the pattern of fat deposition in males, likely through its impact on sex hormones. In males, lipoprotein lipase activity is higher in abdominal adipose tissues, and testosterone inhibits its activity in femoral subcutaneous fat, resulting in the android physique typically observed in males. At least 25% of males with T2DM have subnormal testosterone levels [42, 43], which may contribute to changes in fat distribution. Studies have shown that hypogonadal men have increased fatty acid storage in the lower body subcutaneous area and higher activity of femoral adipose tissue acyl-CoA synthetase, indicating that chronic testosterone deficiency in males with diabetes may lead to fat redistribution via adipocyte enzymes [44]. Moreover, previous research demonstrated that males with diabetes tend to have higher estradiol levels than non-diabetic counterparts [45–47]. Elevated estradiol and lower testosterone levels may contribute to the gynoid body morphology and decreasing A/G ratio observed in males with diabetes. Further investigations are necessary to explore the role and mechanisms of hormone-mediated fat redistribution in males with diabetes, as the change in preferential fat accumulation in males may reflect the progression of DM and be associated with DR. On the other hand, remarkable adipose redistribution following menopause in females might attenuate the association between the A/G ratio and the duration of DM. Therefore, it is reasonable to understand that males with diabetes who have a higher A/G ratio are less likely to have DR, and direct measurement of regional fat may offer a more accurate assessment of retinopathy risk.

This study presents a groundbreaking finding, showing that the DXA-measured A/G ratio, a comprehensive index of regional body fat distribution, served as an independent marker for DR. It is argued that relying solely on BMI or WHtR is insufficient to assess or manage the microvascular risk associated with increased adiposity in the population with diabetes. Current analysis suggests that the fat deposition pattern index, A/G ratio, serves as an independent and superior discriminator compared to anthropometric obesity indices. Based on the compelling evidence from the NHANES, this study strongly advocates for fat distribution to be considered as a vital sign in clinical practice.

This study has several limitations that should be acknowledged. First, due to the limited proportion of participants with T1DM, the findings primarily apply to the T2DM population. Second, the information on DR was extracted from diabetes questionnaires. The methodology used in this study was aligned with previous studies [33–36] to ensure consistency and comparability. Third, being a cross-sectional study, it is challenging to establish cause-and-effect relationships; nevertheless, the findings provide valuable insights for clinicians in managing diabetic complications. Fourth, it is important to note that only the 2003–2006 cycles of NHANES administered DXA to participants aged 60 and older. Among 1 517 participants in the study population, 375 were aged 60 and above, representing a weighted estimate of 1 470 182 individuals. The weighted calculations of the complex sampling strategy and the adjustment for age in multivariable logistic regression analysis mitigate the impact on the representativity of the findings. On the positive side, a significant strength of this study is its representative sample size. NHANES provides generalizable data on a multiethnic population that is nationally representative of the U.S. Moreover, the biochemical data and questionnaire responses were homogeneously collected by trained personnel, enhancing the reliability of the study’s data collection process.

## Conclusion

The novel finding of an association between higher A/G ratio and the presence of DR, independent of BMI and WHtR, underscores the importance of fat distribution as a critical factor in DR. This study highlights that imaging-based fat distribution serves as a more clinically significant marker among individuals with diabetes. Furthermore, it suggests that considering how fat is distributed in the body could be crucial in assessing the risk of retinopathy in the population with diabetes. Further research is warranted to gain a deeper understanding of the mechanisms behind this relationship and its implications for diabetes management and risk assessment.

### Supplementary information

table S1

table S2

table S3

table S4

## Data Availability

Data described in the manuscript and analytic code will be made available upon request pending.
