# Supplementary material for: Imaging-based body fat distribution and diabetic retinopathy in general US population with diabetes: an NHANES analysis (2003–2006 and 2011–2018)
Source: Nutr Diabetes. 2024 Jul 14;14:53. doi: 10.1038/s41387-024-00308-z (PMC11247072; doi:10.1038/s41387-024-00308-z)
Supplement: Supplementary file 1 — table S1 [file 41387_2024_308_MOESM1_ESM.docx]

Table S1: Multivariable associations between BMI and waist-to-height ratio with the presence of diabetic retinopathy in the population with type 2 diabetes and stratified by sex.

|  | **Prevalence** |  | **Overall OR (95% CI)** |  | **Male OR (95% CI)** |  | **Female OR (95% CI)** |
| --- | --- | --- | --- | --- | --- | --- | --- |
| **BMI level** |  |  |  |  |  |  |  |
| **<25** | 23.6% |  | 1 [Reference] |  | 1 [Reference] |  | 1 [Reference] |
| **>=25-<30** | 20.9% |  | 0.916 (0.507, 1.652) |  | 1.271 (0.587, 2.756) |  | 0.455 (0.213, 0.973) |
| **>=30** | 18.9% |  | 0.964 (0.538, 1.726) |  | 1.116 (0.529, 2.356) |  | 0.679 (0.298, 1.547) |
| ***P* for trend** |  |  | 0.986 |  | 0.948 |  | 0.766 |
| **BMI tertiles by ethnicity** |  |  |  |  |  |  |  |
| **Tertile 1** | 24.1% |  | 1 [Reference] |  | 1 [Reference] |  | 1 [Reference] |
| **Tertile 2** | 18.1% |  | 0.822 (0.538, 1.254) |  | 0.776 (0.429, 1.406) |  | 0.907 (0.454, 1.810) |
| **Tertile 3** | 18.0% |  | 0.839 (0.521, 1.349) |  | 0.733 (0.388, 1.388) |  | 0.913 (0.461, 1.809) |
| ***P* for trend** |  |  | 0.456 |  | 0.314 |  | 0.804 |
| **BMI**  **(Per unit increase)** |  |  | 1.001 (0.973, 1.031) |  | 0.995 (0.956, 1.036) |  | 1.005 (0.966, 1.045) |
|  |  |  |  |  |  |  |  |
| **WHtR level** |  |  |  |  |  |  |  |
| **Tertile 1 (<0.61)** | 21.5% |  | 1 [Reference] |  | 1 [Reference] |  | 1 [Reference] |
| **Tertile 2 (0.61-0.69)** | 19.2% |  | 1.120 (0.715, 1.755) |  | 1.113 (0.631, 1.963) |  | 1.158 (0.477, 2.810) |
| **Tertile 3 (>=0.69)** | 18.6% |  | 1.039 (0.651, 1.659) |  | 0.950 (0.468, 1.925) |  | 1.168 (0.511, 2.671) |
| ***P* for trend** |  |  | 0.852 |  | 0.954 |  | 0.717 |
| **WHtR tertiles by ethnicity** |  |  |  |  |  |  |  |
| **Tertile 1** | 21.4% |  | 1 [Reference] |  | 1 [Reference] |  | 1 [Reference] |
| **Tertile 2** | 18.9% |  | 1.084 (0.707, 1.664) |  | 1.094 (0.628, 1.904) |  | 1.158 (0.516, 2.601) |
| **Tertile 3** | 19.1% |  | 1.035 (0.652, 1.643) |  | 0.887 (0.447, 1.757) |  | 1.233 (0.546, 2.788) |
| ***P* for trend** |  |  | 0.876 |  | 0.794 |  | 0.613 |
| **WHtR**  **(Per 0.1-unit increase)** |  |  | 1.006 (0.814, 1.243) |  | 1.000 (0.748, 1.336) |  | 1.015 (0.734, 1.403) |

Abbreviations: BMI, body mass index; WHtR, waist-to-height ratio; OR, odds ratio; CI, confidence interval.

Adjusted for sex, age, race/ethnicity, diabetes duration, hemoglobin A1c level, blood pressure level, non-high-density lipoprotein cholesterol level.

Stratified models are adjusted for covariates not stratified on.
