# Supplementary material for: Imaging-based body fat distribution and diabetic retinopathy in general US population with diabetes: an NHANES analysis (2003–2006 and 2011–2018)
Source: Nutr Diabetes. 2024 Jul 14;14:53. doi: 10.1038/s41387-024-00308-z (PMC11247072; doi:10.1038/s41387-024-00308-z)
Supplement: Supplementary file 2 — table S2 [file 41387_2024_308_MOESM2_ESM.docx]

Table S2: Multivariable associations between A/G ratio and the presence of diabetic retinopathy in the population with type 2 diabetes

|  |  |  |  | **Odds Ratio (95% CI)** | | |
| --- | --- | --- | --- | --- | --- | --- |
| **A/G ratio** |  | **Prevalence (%)** |  | **Model 1** ^a^ | **Model 2** ^b^ | **Model 3** ^c^ |
| **Tertiles categorized by overall population** |  |  |  |  |  |  |
| **1.0-1.2** |  | 21.2% |  | 1 [Reference] | 1 [Reference] | 1 [Reference] |
| **< 1.0** |  | 22.2% |  | 0.805 (0.479, 1.354) | 0.824 (0.482, 1.410) | 0.870 (0.509, 1.489) |
| **≥ 1.2** |  | 17.6% |  | 0.562 (0.369, 0.855) | 0.564 (0.371, 0.857) | 0.583 (0.376, 0.904) |
| ***P* for trend** |  |  |  | 0.063 | 0.047 | 0.046 |
| **Tertiles categorized by ethnicity** |  |  |  |  |  |  |
| **Tertile 2** |  | 21.7% |  | 1 [Reference] | 1 [Reference] | 1 [Reference] |
| **Tertile 1** |  | 21.2% |  | 0.761 (0.447, 1,296) | 0.770 (0.445, 1.332) | 0.810 (0.467, 1.404) |
| **Tertile 3** |  | 17.8% |  | 0.567 (0.367, 0.877) | 0.570 (0.369, 0.881) | 0.591 (0.375, 0.932) |
| ***P* for trend** |  |  |  | 0.109 | 0.089 | 0.087 |
| **A/G ratio**  **(per 0.1-unit increase)** |  |  |  | 0.934 (0.834, 1.047) | 0.926 (0.821, 1.044) | 0.922 (0.810, 1.049) |

Abbreviations: A/G ratio, android to gynoid fat ratio; OR, odds ratio; CI, confidence interval.

^a^ Model 1: Adjusted for sex, age, race/ethnicity, diabetes duration, hemoglobin A1c level, blood pressure level, non-high-density lipoprotein cholesterol level.

^b^ Model 2: Model 1+body mass index.

^c^ Model 3: Model 1+waist-to-height ratio.
