# Supplementary material for: Imaging-based body fat distribution and diabetic retinopathy in general US population with diabetes: an NHANES analysis (2003–2006 and 2011–2018)
Source: Nutr Diabetes. 2024 Jul 14;14:53. doi: 10.1038/s41387-024-00308-z (PMC11247072; doi:10.1038/s41387-024-00308-z)
Supplement: Supplementary file 3 — table S3 [file 41387_2024_308_MOESM3_ESM.docx]

Table S3: Multivariable associations between A/G ratio and the presence of diabetic retinopathy in the population with type 2 diabetes stratified by sex.

|  | **Odds Ratio (95% CI)** | | | | | |
| --- | --- | --- | --- | --- | --- | --- |
|  | **Male** | | | **Female** | | |
| **A/G ratio** | **Model 1** ^a^ | **Model 2** ^b^ | **Model 3** ^c^ | **Model 1** ^a^ | **Model 2** ^b^ | **Model 3** ^c^ |
| **Tertiles categorized by overall population** |  |  |  |  |  |  |
| **1.0-1.2** | 1 [Reference] | 1 [Reference] | 1 [Reference] | 1 [Reference] | 1 [Reference] | 1 [Reference] |
| **<1.0** | 0.576  (0.236, 1.409) | 0.572  (0.224, 1.456) | 0.647  (0.254, 1.644) | 0.944  (0.512, 1.741) | 0.970  (0.518, 1.817) | 0.988  (0.545, 1.792) |
| **≥1.2** | 0.487  (0.289, 0.822) | 0.492  (0.288, 0.841) | 0.520  (0.300, 0.900) | 0.764  (0.329, 1.772) | 0.768  (0.328, 1.801) | 0.747  (0.315, 1.771) |
| ***P* for trend** | 0.070 | 0.088 | 0.092 | 0.738 | 0.654 | 0.553 |
| **Tertiles categorized by ethnicity** |  |  |  |  |  |  |
| **Tertile 2** | 1 [Reference] | 1 [Reference] | 1 [Reference] | 1 [Reference] | 1 [Reference] | 1 [Reference] |
| **Tertile 1** | 0.562  (0.235, 1.339) | 0.553  (0.224, 1.363) | 0.623  (0.253, 1.538) | 0.875  (0.463, 1.656) | 0.889  (0.462, 1.710) | 0.897  (0.479, 1.680) |
| **Tertile 3** | 0.482  (0.278, 0.839) | 0.488  (0.278, 0.856) | 0.517  (0.289, 0.926) | 0.762  (0.334, 1.741) | 0.765  (0.330, 1.772) | 0.746  (0.320, 1.742) |
| ***P* for trend** | 0.096 | 0.118 | 0.123 | 0.865 | 0.792 | 0.694 |
| **A/G ratio**  **(per 0.1-unit increase)** | 0.938  (0.805, 1.094) | 0.938  (0.795, 1.108) | 0.939  (0.792, 1.114) | 0.935  (0.766, 1.142) | 0.918  (0.751, 1.122) | 0.893  (0.724, 1.102) |

Abbreviations: A/G ratio, android to gynoid fat ratio; OR, odds ratio; CI, confidence interval.

^a^ Model 1: Adjusted for age, race/ethnicity, diabetes duration, hemoglobin A1c level, blood pressure level, non-high-density lipoprotein cholesterol level.

^b^ Model 2: Model 1+body mass index.

^c^ Model 3: Model 1+waist-to-height ratio.
