# Supplementary material for: Imaging-based body fat distribution and diabetic retinopathy in general US population with diabetes: an NHANES analysis (2003–2006 and 2011–2018)
Source: Nutr Diabetes. 2024 Jul 14;14:53. doi: 10.1038/s41387-024-00308-z (PMC11247072; doi:10.1038/s41387-024-00308-z)
Supplement: Supplementary file 4 — table S4 [file 41387_2024_308_MOESM4_ESM.docx]

Table S4: Multivariable associations between A/G ratio and the presence of diabetic retinopathy in the population with type 2 diabetes and stratified by sex.

|  | **Odds Ratio (95% CI)** | | | | | |
| --- | --- | --- | --- | --- | --- | --- |
| **A/G ratio** | **Overall** |  | **Male** |  | **Female** |  |
| **Tertiles categorized by overall population** |  |  |  |  |  |  |
| **1.0-1.2** | 1 [Reference] |  | 1 [Reference] |  | 1 [Reference] |  |
| **<1.0** | 0.774 (0.455, 1.316) |  | 0.546 (0.200, 1.492) |  | 0.902 (0.489, 1.666) |  |
| **≥1.2** | 0.594 (0.384, 0.920) |  | 0.539 (0.309, 0.942) |  | 0.704 (0.304, 1.633) |  |
| ***P* for trend** | 0.151 |  | 0.216 |  | 0.715 |  |
| **Tertiles categorized by ethnicity** |  |  |  |  |  |  |
| **Tertile 2** | 1 [Reference] |  | 1 [Reference] |  | 1 [Reference] |  |
| **Tertile 1** | 0.727 (0.422, 1.251) |  | 0.524 (0.197, 1.393) |  | 0.835 (0.443, 1.573) |  |
| **Tertile 3** | 0.584 (0.373, 0.915) |  | 0.520 (0.289, 0.934) |  | 0.690 (0.304, 1.564) |  |
| ***P* for trend** | 0.202 |  | 0.242 |  | 0.810 |  |
| **A/G ratio**  **(per 0.1-unit increase)** | 0.956 (0.855, 1.069) |  | 0.967 (0.835, 1.120) |  | 0.949 (0.783, 1.150) |  |

Abbreviations: A/G ratio, android to gynoid fat ratio; OR, odds ratio; CI, confidence interval.

Adjusted for age, sex, race/ethnicity, diabetes duration, hemoglobin A1c level, blood pressure level, non-high-density lipoprotein cholesterol level, and insulin use.

Stratified models are adjusted for covariates not stratified on.
